# Determination of the residual efficacy of carbamate and organophosphate insecticides used for indoor residual spraying for malaria control in Ethiopia

**DOI:** 10.1186/s12936-017-2122-3

**Published:** 2017-11-21

**Authors:** Delenasaw Yewhalaw, Meshesha Balkew, Josephat Shililu, Sultan Suleman, Alemayehu Getachew, Gedeon Ashenbo, Sheleme Chibsa, Gunawardena Dissanayake, Kristen George, Dereje Dengela, Yemane Ye-Ebiyo, Seth R. Irish

**Affiliations:** 1 Tropical and Infectious Diseases Research Center, Jimma University, Jimma, Ethiopia; 2 Department of Medical Laboratory Sciences and Pathology, College of Health Sciences, Jimma University, Jimma, Ethiopia; 3 Aklilu Lemma Institute of Pathobiology, Addis Ababa University, Addis Ababa, Ethiopia; 4 The President’s Malaria Initiative Africa Indoor Residual Spraying Project, Abt Associates, Gerji Road, Sami Building, 1st Floor, Addis Ababa, Ethiopia; 5 Department of Pharmacy, College of Health Sciences, Jimma University, Jimma, Ethiopia; 6 U.S. Agency for International Development (USAID), Entoto Street, Addis Ababa, Ethiopia; 7 President’s Malaria Initiative, Bureau for Global Health, Office of Infectious Disease, United States Agency for International Development, 1300 Pennsylvania Ave NW, Washington, DC 20523 USA; 8 The President’s Malaria Initiative Africa Indoor Residual Spraying Project, Abt Associates, 4550 Montgomery Ave., Suite 800 North, Bethesda, MD 20814 USA; 9 The US President’s Malaria Initiative and Entomology Branch, Centers for Disease Control and Prevention, 1600 Clifton Road, Atlanta, GA 30329-4027 USA

**Keywords:** Indoor residual spraying, Bendiocarb, Propoxur, Pirimiphos-methyl, Residual efficacy, Ethiopia

## Abstract

**Background:**

Indoor residual spraying is one of the key vector control interventions for malaria control in Ethiopia. As malaria transmission is seasonal in most parts of Ethiopia, a single round of spraying can usually provide effective protection against malaria, provided the insecticide remains effective over the entire malaria transmission season. This experiment was designed to evaluate the residual efficacy of bendiocarb, pirimiphos-methyl, and two doses of propoxur on four different wall surfaces (rough mud, smooth mud, dung, and paint). Filter papers affixed to wall surfaces prior to spraying were analyzed to determine the actual concentration applied. Cone bioassays using a susceptible *Anopheles arabiensis* strain were done monthly to determine the time for which insecticides were effective in killing mosquitoes.

**Results:**

The mean insecticide dosage of bendiocarb applied to walls was 486 mg/m^2^ (target 400/mg). This treatment lasted 1 month or less on rough mud, smooth mud, and dung, but 4 months on painted surfaces. Pirimiphos-methyl was applied at 1854 mg/m^2^ (target 1000 mg/m^2^), and lasted between 4 and 6 months on all wall surfaces. Propoxur with a target dose of 1000 mg/m^2^ was applied at 320 mg/m^2^, and lasted 2 months or less on all surfaces, except painted surfaces (4 months). Propoxur with a target dose of 2000 mg/m^2^, was applied at 638 mg/m^2^, and lasted 3 months on rough mud, but considerably longer (5–7 months) on the other substrates.

**Conclusions:**

It would appear that the higher dose of propoxur and pirimiphos-methyl correspond best to the Ethiopian transmission season, although interactions between insecticide and the substrate should be taken into account as well. However, the insecticide quantification revealed that the dosages actually applied differed considerably from the target dosages, even though care was taken in the mixing of insecticide formulations and spraying of the walls. It is unclear whether this variability is due to initial concentrations of insecticides, poor application, or other factors. Further work is needed to ensure that target doses are correctly applied, both operationally and in insecticide evaluations.

**Electronic supplementary material:**

The online version of this article (10.1186/s12936-017-2122-3) contains supplementary material, which is available to authorized users.

## Background

In addition to the distribution and use of insecticide-treated nets (ITNs), indoor residual spraying (IRS) is one of the most effective methods of malaria vector control in areas where mosquitoes are endophilic. The benefits of IRS include a continuous killing effect, reducing the abundance and longevity of the vectors, reduced need for continued compliance after the initial spray, and the fact that some formulations can last for the entire transmission season, providing protection when it is most needed.

Currently, only four classes of insecticide are recommended for IRS: organochlorines, pyrethroids, carbamates, and organophosphates [1]. In many places in Ethiopia, resistance to the organochlorine DDT has developed to the point that almost no killing effect is noticed [2, 3].

Pyrethroids are cheap and long-lasting, but as they are the only class recommended for insecticide treated nets, there are serious concerns about using pyrethroids for IRS when other options are available [4]. Moreover, resistance to pyrethroids is also widespread due to shared mechanisms with organochlorines (*kdr*) and widespread use of this class in ITNs [5]. This would leave only carbamates and organophosphates as viable alternatives for IRS, which is challenging, as these insecticides are more expensive and also may share a resistance mechanism (insensitive acetylcholinesterase), which increases the difficulties for effective insecticide rotation to manage resistance.

Indoor residual spraying is a long-standing practice in Ethiopia. DDT was used from the time of the Malaria Eradication Programme (late 1950s) until 2009, deltamethrin was used from 2010 to 2012, and bendiocarb has been applied since 2011. Propoxur has also been in use since 2012, and limited pirimiphos-methyl has been used since 2015. Insecticide resistance monitoring is an important activity for the Federal Ministry of Health, and is used to guide insecticide choice (as described in its insecticide resistance monitoring and management strategic plan). Another important consideration is the persistence of the insecticides, as an insecticide which requires more than one application per year would considerably increase the cost of the IRS programme.

To this end, the residual efficacy of propoxur and bendiocarb were evaluated by the President’s Malaria Initiative-funded Ethiopia Africa Indoor Residual Spraying (AIRS) project [6]. The residual efficacy of the two insecticides were compared over a period of 6 months on several substrates, and it was found that bendiocarb (400 mg/m^2^) had a short residual life on porous surfaces, whereas propoxur (2000 mg/m^2^) resulted in over 80% mortality of mosquitoes in 30 min cone tests for at least 6 months on all surfaces tested [6].

The considerable differences in efficacy over time shown by different insecticides on different substrates are not surprising. Djènontin et al. [7] found bendiocarb (WP 80 W Ficam, target dose: 400 mg/m^2^) to result in at least 80% mortality for 13 weeks on teak wood, 7 weeks on cement, and 6 weeks on red clay. Tangena et al. [8] found bendiocarb (WP 80 W Ficam, target dose: 400 mg/m^2^) to result in mortality higher than 80% for at least 5 months on mud walls, perhaps explained by the fact that the actual dose was closer to 1000 mg/m^2^. Etang et al. [9] found nearly 100% mortality for 13 weeks when bendiocarb WP (target dose 400 mg/m^2^) was applied to concrete and wood, but mortality was only 20% after 13 weeks on mud. The large variation in the results indicates that the substrate is important and local testing is necessary to have an accurate expectation of residual efficacy.

The aim of this study was to compare the residual efficacy of three insecticides (bendiocarb 400 mg/m^2^, pirimiphos-methyl 1000 mg/m^2^, propoxur 2000 mg/m^2^) at dosages currently in use for Ethiopia’s spray programme, as well as to evaluate a lower dosage of propoxur (1000 mg/m^2^) which is within WHO-recommended dosages for propoxur [1], to see if savings might be made without compromising spray effectiveness. These treatments were applied to four common wall surface types (rough mud, smooth mud, dung, paint) in huts to ensure that conclusions from this study would be valid for the common wall surface types of houses found throughout Ethiopia.

## Methods

### Study sites

The AdamiTullu-Judo-Kombolcha district is situated in East Shoa (7°56′N 38°43′E), approximately 1643 metres above sea level. It is home to Lake Ziway which is a source of fishing and irrigation for farming, the main occupations in the area. The district population was 141,405 in the last national census [10]. One set of 10 huts was built in Gerbi-Widena-Boremo kebele, close to Ziway town. For simplicity, this site is referred to hereafter as Ziway.

The second group of 10 huts was constructed at the Tropical and Infectious Diseases Research Center (TIDRC) in Sekoru district. Sekoru is situated 102 km from Jimma, the largest city in southwestern Ethiopia, with a population of 207,573. Sekoru lies at 1780 m above sea level.

### Treatments

The treatments compared in this study included:

1. Bendiocarb 400 mg/m^2^: Ficam VC (WP 80%, Bayer S.A.S., Lyon, France)
2. Propoxur 1000 mg/m^2^: Ethio propoxur 50% WP (Adami-Tulu Pesticides Processing S.Co., Ethiopia)
3. Propoxur 2000 mg/m^2^: Ethio propoxur 50% WP (Adami-Tulu Pesticides Processing S.Co., Ethiopia)
4. Pirimiphos-methyl 1000 mg/m^2^: Actellic 300CS (Syngenta AG, Basel, Switzerland)
5. Control (water)

An informal survey of wall surfaces of local houses was made in February 2015 to determine the most common wall surface types in and around Jimma and Ziway by two of the investigators (SI, SC). The most common wall surfaces were a rough plastering of mud (rough mud), a second smoother layer of mud over rough mud (smooth mud), and smooth mud covered with lime and then painted (paint). A fourth wall treatment (dung) was added as this is a common wall treatment in other parts of Ethiopia. In summary, the four types of wall surface tested were:

1. Mud (first layer, somewhat rough application) (Fig. 1a, left side)

**Fig. 1:**
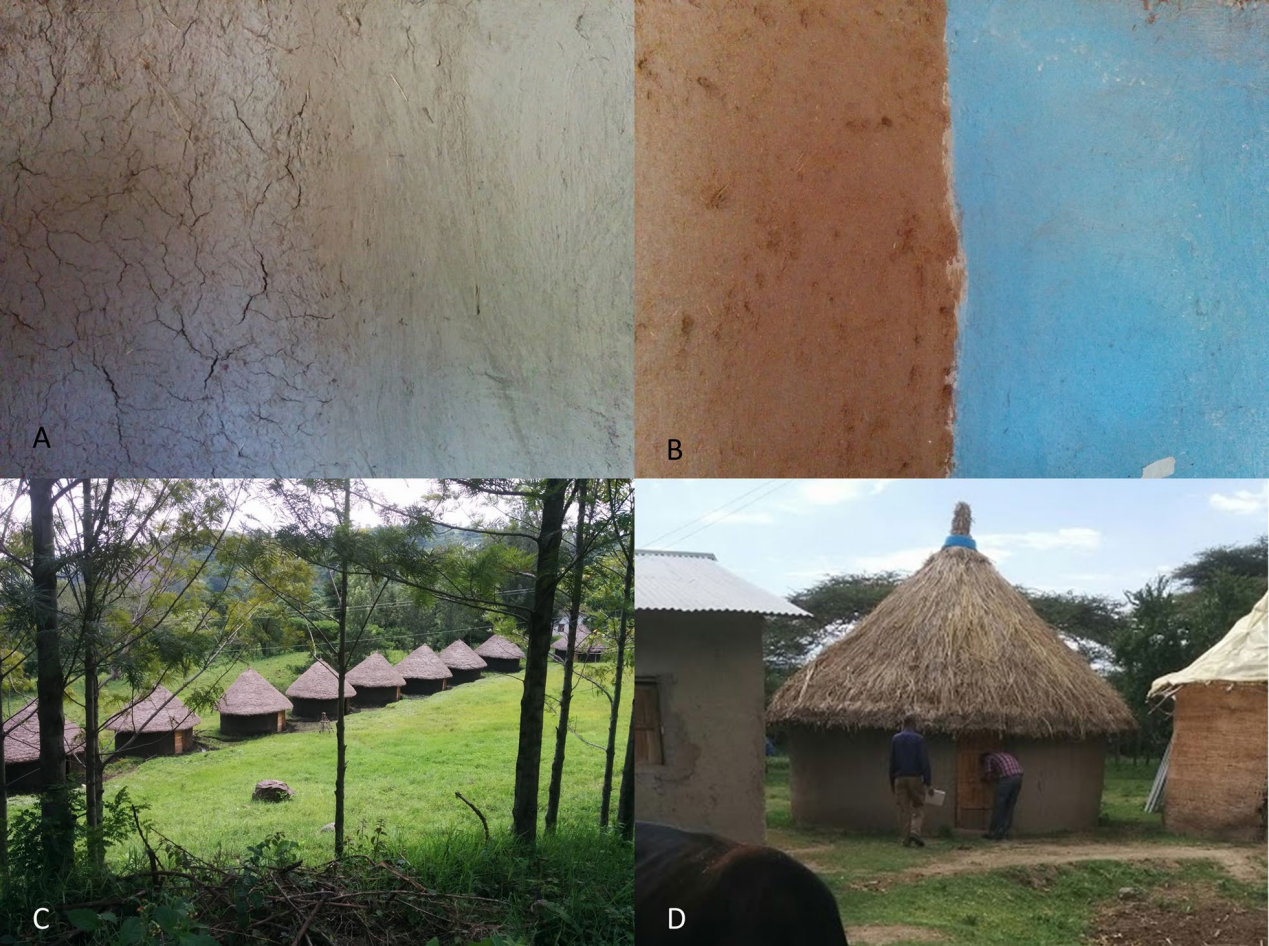
**a** Rough (left) and smooth (right) mud walls, **b** dung (left) and painted (right) walls, **c** huts in Sekoru **d** example of the huts in Ziway
2. Mud (second layer, very smooth application) (Fig. 1a, right side)
3. Dung (a layer of dung over the second smooth layer of mud) (Fig. 1b, left side)
4. Paint (a layer of paint over a layer of lime, applied over second smooth layer of mud) (Fig. 1b, right side)

As types of mud may vary based on the type of soil and preparation of mud for plastering in different parts of the country, this study was conducted in two locations in Oromia (Ziway and Sekoru).

In each location 10 simple test huts were constructed (Fig. 1c and d), allowing for two replicates of each insecticide treatment in each site. In Sekoru, the huts were of traditional “tukul” type which are circular huts, constructed using a wattle and daub technique, thatched roof and walls with wooden frame of eucalyptus, plastered with mud. In Ziway the huts were also thatched roof but the walls were made of mud bricks plastered with mud. Huts had no windows. The interior diameter of the huts was approximately 4 m and the height of the walls was between 2 and 3 m. The interiors of huts were divided into four sections and each section was covered with one of the four surfaces treatments. Each hut was sprayed with a single treatment by an experienced spray operator; non-stop starting from the door and moving clockwise to cover the entire wall surface of the hut.

### Assessment of insecticide concentration

To assess the quality of the spray, four filter papers (Whatman No. 1) were placed on the walls of each hut, one on each surface type. The papers were placed in areas where spray overlap was unlikely, to ensure that the quantity of insecticide was not overestimated. The locations of the papers were marked with chalk to ensure subsequent bioassays were not conducted in locations where papers prevented the spray from reaching the wall surfaces. The filter papers were collected after spray, wrapped in aluminum foil labelled by site hut number, insecticide formulation, type of surface and date of spraying, and kept individually at 4 °C in a refrigerator in the laboratory until processing. The insecticide concentration was analyzed using High Performance Liquid Chromatography, using the methods previously described [8, 11], at the Drug Quality Control Laboratory at Jimma University, Ethiopia.

### Assessment of residual activity

World Health Organization cone bioassays were used to monitor residual efficacy of each insecticide on sprayed walls. Mosquitoes from insectaries (see below) were transported to the huts in cages covered with damp towels to maintain humidity and temperature. At the trial site batches of ten female mosquitoes were transferred into paper cups covered with netting. Cotton pads soaked in 10% sugar solution were placed on top of each cup. On each surface type in each hut, three cones were affixed to indoor walls at three different heights: high (40 cm below the roof), middle and low (40 cm high from the floor) to evaluate the persistence of insecticides [12]. Mosquitoes from each paper cup were introduced into the cones using mouth aspirators (a separate aspirator was used for each insecticide and dose). Cones were attached to the walls using small nails. The location of each cone was marked on the wall to ensure that locations were not retested. After 30 min of exposure the mosquitoes were removed from the cones and returned to the paper cups, which were then kept in a wooden box covered with a moist towel and mortality was recorded after 24 h. A mosquito was considered as alive if it was able to fly. If control mortality was over 10%, the bioassays were repeated.

Bioassays were conducted in each house after 48–72 h (post-spray) and once per month for 6 months. Ideally, all bioassays were to be conducted the same day, but as this was not logistically possible, all bioassays were conducted within 7 days of each other. Treatment-substrate combinations still effective at 6 months were tested at 7 months.

### Mosquitoes

Teams in Ziway and Sekoru used a strain of *Anopheles arabiensis* colonized from mosquitoes collected near Adama, Ethiopia. This strain is known to be susceptible to the organophosphate and carbamate insecticides used in this study. Susceptibility was confirmed using WHO tube tests prior to use in the experiments. Two to 5 day-old female mosquitoes fed ad libitum with sugar solution (10%) were used for the bioassays.

### Analysis

The mean percentage mortality of mosquitoes was calculated for each site, insecticide, and substrate using Stata 14 (College Station, TX). The WHO considers a mortality of 80% the cutoff for effective insecticidal effect of indoor residual spraying [12], so the number of months the treatments were effective was calculated using this criterion. The influence of the variables: study site, time since spraying, house (replicate), insecticide treatment, substrate type (wall surface), the position on the wall, and an interaction between the insecticide and substrate on the mortality of mosquitoes in cone bioassays were analyzed using a logistic regression model, using backwards selection of variables until all factors were significantly associated (p ≤ 0.05) with the mortality of mosquitoes.

## Results

### Assessment of insecticide concentration

A summary of the calculated doses applied to walls, as determined from the filter papers, is provided in Table 1. The bendiocarb treatment was an average of 22% more than the target dose. The other treatments varied considerably from the target dosages. The mean dosage for pirimiphos-methyl was calculated to be 1854 mg/m^2^, 85% higher than the target dosage of 1000 mg/m^2^. The mean dosage of propoxur for houses targeted with 1000 mg/m^2^ was actually 322 mg/m^2^, only 32% of the target dosage. The mean dosage of propoxur for houses targeted with 2000 mg/m^2^ was actually 656 mg/m^2^, only 33% of the target dose. For both sites, the same pattern of under- or over-dosing was found (Table 1).

**Table 1:** Target and actual doses of different insecticide formulations analysed by high-performance liquid chromatography of extracts from filter papers placed on different wall surfaces at the time of spraying

Insecticide	Target dose (mg/m^2^)	Site	Samples tested	Mean (95% CI)	Mean by insecticide (95% CI)
Bendiocarb	400	Sekoru	8	465 (339–590)	486 (417–555)
		Ziway	8	506 (414–599)	
Pirimiphos methyl	1000	Sekoru	8	1967 (1725–2210)	1854 (1663–2045)
		Ziway	8	1741 (1404–2077)	
Propoxur	1000	Sekoru	8	357 (323–390)	322 (290–354)
		Ziway	8	286 (239–334)	
Propoxur	2000	Sekoru	8	753 (647–860)	656 (574–738)
		Ziway	8	559 (465–653)	

### Assessment of residual efficacy

**Fig. 2:**
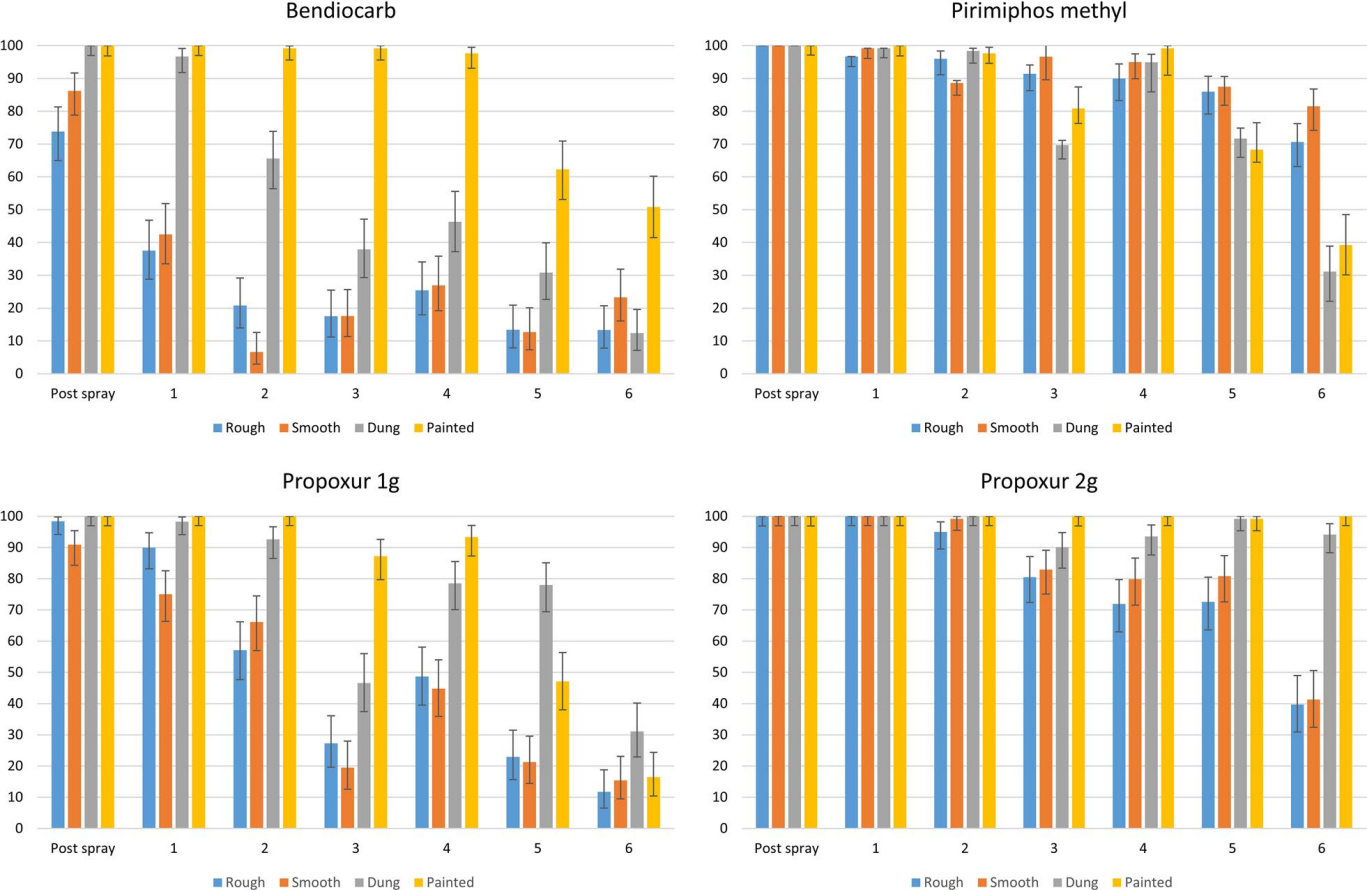
Mortality (and 95% confidence intervals) of *Anopheles arabiensis* in 30 min cone bioassays on different wall substrates treated with four insecticides

Over the course of the study, 19,369 *An. arabiensis* were used for the cone bioassays. The control mortality for each site ranged from 0 to 5.98%. The control mortality was only over 5% in Sekoru during the first month. As a result Abbott’s correction [13], which is commonly used in such situations when describing summary data, was not used, so that the other variables could be considered in the logistic regression. The variables of site (Sekoru or Ziway), hut, insecticide treatment, time since spraying, wall substrate, position of the cone on the wall (high, middle or low), and the interaction of the insecticide with the substrate were used to develop a logistic regression model. The position of the cone on the wall and the hut were not significantly associated with the mortality of mosquitoes in the cone bioassays, but all other variables were significantly associated (p < 0.05). The mortality of the mosquitoes in cone bioassays for each insecticide, on each substrate, for each time period is shown in Fig. 2. The site specific mortality rates and data are provided in Additional file 1: Figure S1, Additional file 2: Figure S2, Additional file 3.

The mortality for bendiocarb was less than 80% within 96 h of treatment on the rough mud surface. Similarly, 1 month after treatment, the mortality of mosquitoes in cones on the smooth mud surface was 60%. The treatment on dung was effective for 1 month, but dropped below 80% at the second month. On painted surfaces, however, bendiocarb treatments remained insecticidal for 4 months, before dropping to 61% mortality on the fifth month.

Pirimiphos-methyl was effective for all substrates for the first 2 months. However, in the third month, the mortality on dung was less than 80% (70%). This decrease seems to be related to a drop in the results from Ziway, with a 53% mortality in the third month. In Sekoru, the mortality on dung remained high (86%). In both sites, the mortality increased in the fourth month, with a combined mortality of 94%, before decreasing in the fifth month. On the smooth mud, pirimiphos-methyl maintained a combined mortality of over 80% for 6 months, before dropping to 63% for Sekoru in the seventh month.

Propoxur at a target dose of 1000 mg/m^2^ was effective for all substrates for the first post-spray bioassays, 48–72 h after spraying. However, in the first month, the mortality of mosquitoes exposed to treated smooth mud was 75%. In the second month, mortality on rough mud was also below 80% (57%). The treatment efficacy lasted for 2 months on dung, but mortality fell to 47% in the third month. The treatment efficacy on painted surfaces lasted for 4 months.

Propoxur at a target dose of 2000 mg/m^2^ was effective on all substrates for at least 3 months. The mortality of the treatment on rough mud fell below 80% after 3 months. The treatment on smooth mud remained effective for 5 months, although the mortality was 79.8% in the fourth month. The mortality remained high for treated dung (6 months) and painted surfaces (7 months).

A summary of the estimated effective time for each treatment as determined by the number of months that the insecticide remained effective in killing at least 80% of mosquitoes is provided in Table 2.

**Table 2:** The estimated duration of residual efficacy of insecticide treatments on different wall surfaces in two sites (Sekoru and Ziway) in Ethiopia, and the combined results, as determined by the number of months that the treatment resulted in greater than 80% mortality in 30 min cone bioassays using a susceptible *Anopheles arabiensis* strain

	Sekoru				Ziway				Combined			
	Bendiocarb (months)	Actellic (months)	Propoxur 1 g (months)	Propoxur 2 g (months)	Bendiocarb (months)	Actellic	Propoxur 1 g (months)	Propoxur 2 g (months)	Bendiocarb (months)	Actellic (months)	Propoxur 1 g (months)	Propoxur 2 g (months)
Rough mud	< 1	5	1	2^b^	< 1	5	1	4	< 1	5	1	3
Smooth mud	< 1	6	1	5	< 1	6	< 1	2	< 1	6	< 1	5^a^
Dung	1	5	4^a^	7	2	4^a^	2^b^	6	1	4^a^	2	7
Painted	4	5	4^a^	7	4	4^a^	4	6	4	4^a^	4	6

^a^Mortality was less than 80% for only 1 month before increasing to more than 80%

^b^Mortality was above 80% in the fifth month

## Discussion

Establishing the residual efficacy of IRS insecticides is operationally important to the National Malaria Control and Elimination Programme (NMCEP) of the Federal Ministry of Health in Ethiopia. The NMCEP has recently developed an Insecticide Resistance Monitoring and Management (IRMM) Strategy that guides the judicious use of insecticides, taking into account the resistance profiles of malaria vectors, but also the transmission season in areas where they are to be used. The evaluation of the residual efficacy of insecticides should also take into account new insecticides as they come to market to ensure that the NMCEP has the most up-to-date information for insecticide choice for IRS.

The challenges in applying the target dose of insecticide to a wall are not new for indoor residual spray programmes. A regular part of the training of spray applicators is done using water in the tanks so that sprayers can apply the correct swath to a wall, moving the spray wand at the appropriate speed and distance from the wall to ensure that the spray is applied correctly. It is known that spray operators in the field may not follow such exact protocols, and as a result numerous quantification kits have been developed to assess the quantity of insecticide on the walls [14–16]. Additionally, in certain programmes, such as the PMI-funded IRS that is conducted in Ethiopia, bioassays are conducted in a number of houses to ensure that a lethal dose has been applied to the walls. Nevertheless, applying an exact dose remains a challenge and it is notable that in numerous studies evaluating IRS efficacy, including this one, this dose has been consistently missed. Pinder et al. [17] evaluated the addition of IRS with DDT to communities with high use of LLINs in The Gambia, which included the quantification of DDT applied to walls. They used filter papers, carefully supervised the spraying targeting a 2000 mg/m^2^ dose, and found a dose of 1690 g/m^2^ in 2010, and a dose of 3270 mg/m^2^ in 2011. Similarly, Tangena et al. [8], also working in The Gambia found application rates of more than double the target dose for bendiocarb (980 mg/m^2^, target dose 400 mg/m^2^), higher than targeted for DDT (3440 mg/m^2^, target dose 2000 mg/m^2^), but much closer to the target dose for pirimiphos-methyl (1120 mg/m^2^, target dose 1000 mg/m^2^). In a recent WHOPES report that evaluated two IRS formulations [18], only 11 of 32 treatments evaluated had filter paper results showing the target dose was within the ± 25% acceptable range. For example, in the evaluation of chlorfenapyr 240SC, the filter paper analysis found more than a double dose was applied for both chlorfenapyr and alphacypermethrin [18]. More worryingly, in many IRS evaluations, the quantification is not made. In the present results, the dose of bendiocarb was 22% higher than the target dose, but the pirimiphos-methyl dose was approaching double the target dose. The propoxur doses were only nearly a third of the target dose, but interestingly, the ratio between them was nearly 1:2. We are not able to account for why the target doses were missed so dramatically and consistently in both locations. The present results are actually the results of the second experiment that were conducted using these huts. The first experiment was cancelled due to concerns about the spray quality (spray width was judged to be incorrect, which was detectable by the visible spray deposits of propoxur 2000 mg/m^2^). At the beginning of the second experiment, the quality of spray was of concern and a number of the investigators (DY, MB, JS, SC) were on site to ensure that the appropriate doses were applied. To prevent such problems a number of steps have been considered or suggested for future experiments involving IRS applications. These include: chemical testing of the initial formulation to ensure that the concentration of active ingredient is present from the beginning, marking of the walls to ensure consistent swath width, weighing the spray tank before and after spraying of each hut to know how much of the mixture was applied in the huts, and testing half of each filter paper to allow for reanalysis if necessary (Additional file 3).

The bendiocarb results were surprising in light of the proximity of the actual dose to the target dose. For both rough and smooth mud, the mortality was below 80% after 1 month, and the mortality on rough mud did not exceed 80% immediately after spraying. While poor results of bendiocarb on these surfaces have been previously reported from Ethiopia [6], these results are of concern. However, the performance of bendiocarb on painted surfaces was considerably better, lasting longer for 4 months.

The killing effect of pirimiphos-methyl applied to walls was considerably better on both mud- and dung-plastered walls, resulting in 5 months of effectiveness on the rough mud and 6  months on smooth mud, although this may be due to the overapplication of the insecticide to the walls. Surprisingly, the pirimiphos-methyl lasted for a shorter period (4 months) on dung and painted wall surfaces. Four to six months insecticide residual efficacy should be sufficient to cover the malaria transmission season in most areas of Ethiopia [19]. These effective durations are within the range of residual efficacy found elsewhere, which range from 2 to 10 months, depending on the substrate [20–24].

The two doses of propoxur resulted in differing results. The propoxur at a target dose of 1000 mg/m^2^ did not last longer than 2 months on any of the treated wall surfaces other than painted surfaces, for which it lasted 4 months. In contrast, the propoxur with a target dose of 2000 mg/m^2^ lasted longer than any other insecticide formulations on dung and painted surfaces, 7 and 6 months, respectively. However, its performance was considerably less on rough mud (3 months) and smooth mud (5 months). In reality, the propoxur with a target does of 2000 mg/m^2^ was closer to the target dose of 1000 mg/m^2^, indicating that propoxur applied at 1000 mg/m^2^ could be an appropriate dose for control of *An. arabiensis* in Ethiopia.

## Conclusions

The difficulties in applying the target dose in indoor residual spraying were evident in this experiment and are surely of importance in regular IRS interventions in Ethiopia and elsewhere. The need for more advanced technologies in spray equipment and high quality training of spray operators may also be necessary. The results presented here indicate that there are insecticide-dose combinations that are appropriate for control of malaria vectors during the transmission season in Ethiopia, but further work should be done to reproduce the data presented here. Nevertheless, when IRS was conducted as we would expect it to be done in the field, the pirimiphos-methyl and propoxur with a target dose of 2000 mg/m^2^ would be expected to remain effective for the malaria transmission season in Ethiopia.

## Additional files

**Additional file 1: Figure S1.** Mortality (and 95% confidence intervals) of *Anopheles arabiensis* in 30 minute cone bioassays on different wall substrates treated with four insecticides in Sekoru.

**Additional file 2: Figure S2.** Mortality (and 95% confidence intervals) of *Anopheles arabiensis* in 30 minute cone bioassays on different wall substrates treated with four insecticides in Ziway.

**Additional file 3.** Excel spreadsheet containing individual chemical analysis results and bioassay data results.

## Abbreviations

AIRS: Africa Indoor Residual Spraying project (Abt Associates)
DDT: dichlorodiphenyltrichloroethane
IRMM: insecticide resistance monitoring and management
IRS: indoor residual spraying
LLIN: long-lasting insecticidal net
NMCEP: National Malaria Control and Elimination Programme
PMI: US President’s Malaria Initiative
WHO: World Health Organization
WP: wettable powder
