# Supplementary figures and images for: Determination of the residual efficacy of carbamate and organophosphate insecticides used for indoor residual spraying for malaria control in Ethiopia
Source: Malar J. 2017 Nov 21;16:471. doi: 10.1186/s12936-017-2122-3 (PMC5697437; doi:10.1186/s12936-017-2122-3)

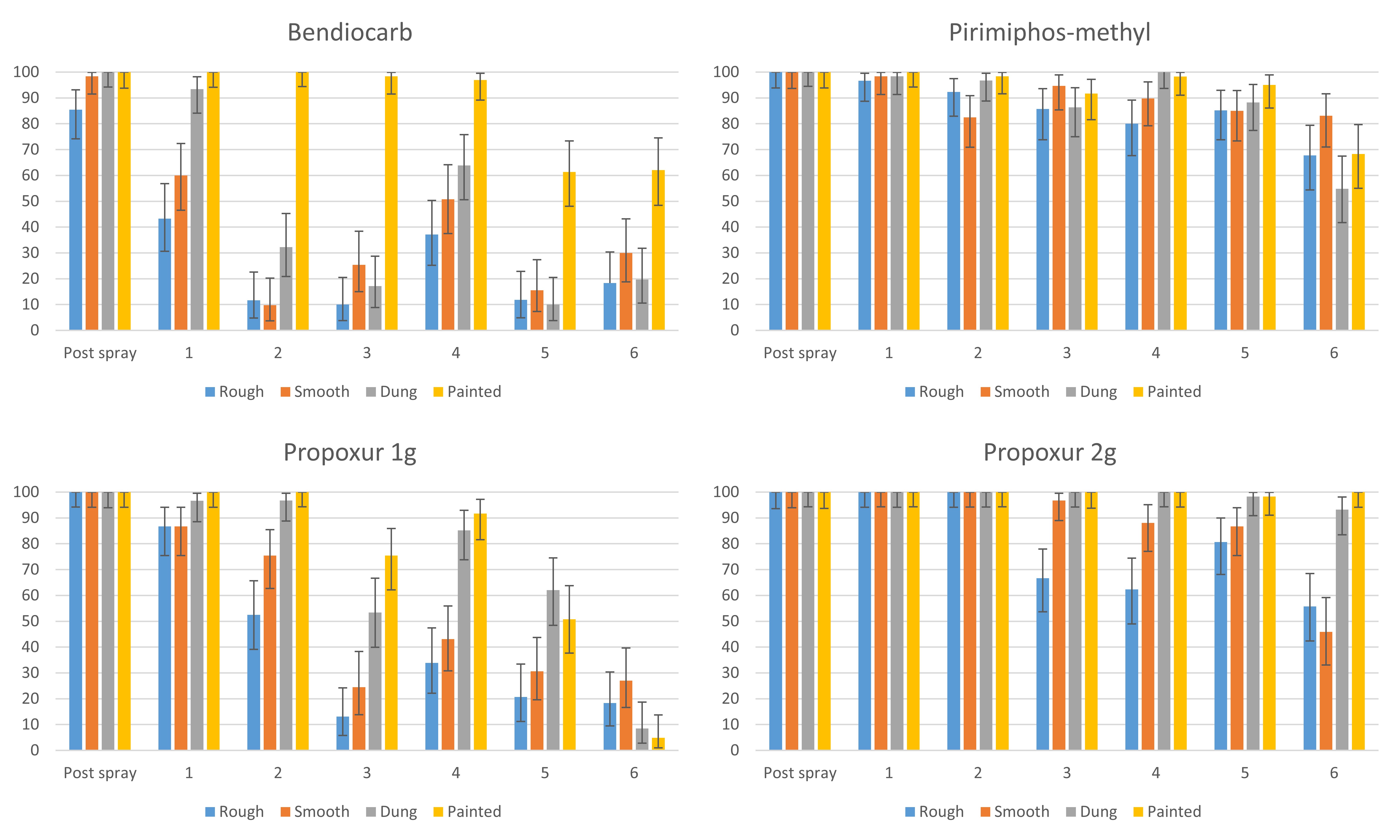

Supplement: Supplementary file 1 — Additional file 1: Figure S1. Mortality (and 95% confidence intervals) of Anopheles arabiensis in 30 minute cone bioassays on different wall substrates treated with four insecticides in Sekoru. [file 12936_2017_2122_MOESM1_ESM.jpg]

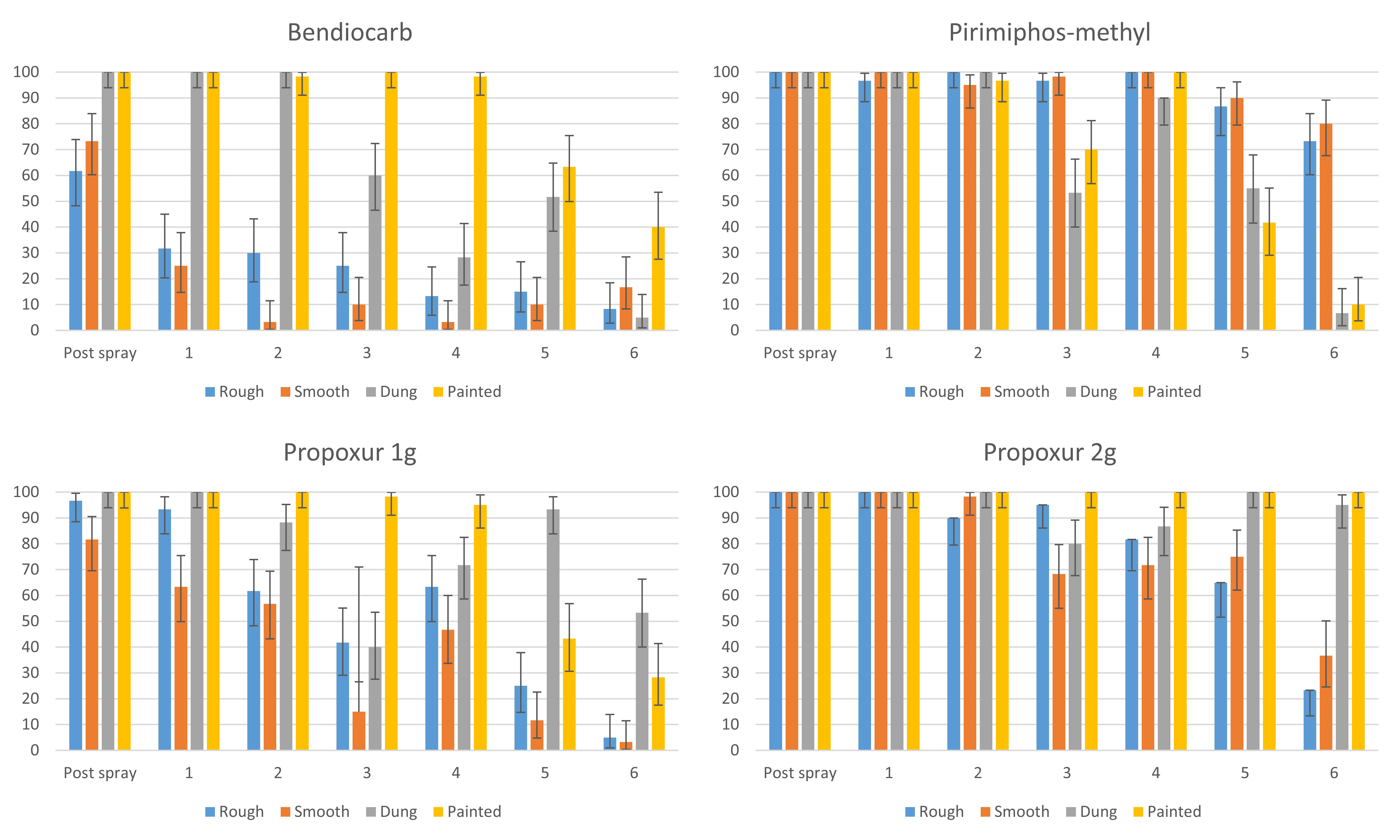

Supplement: Supplementary file 2 — Additional file 2: Figure S2. Mortality (and 95% confidence intervals) of Anopheles arabiensis in 30 minute cone bioassays on different wall substrates treated with four insecticides in Ziway. [file 12936_2017_2122_MOESM2_ESM.jpg]
